# Diversity of Antarctic sea ice and under-ice seawater RNA viruses

**DOI:** 10.1128/aem.01472-26

**Published:** 2026-08-26

**Authors:** Shujuan Sun, Kaiyang Zheng, Yue Su, Yantao Liang, Miaolan Wu, Hao Yu, Jianhua Sun, Wei Wang, Andrew Martin, Fraser Kennedy, Ken Ryan, Chen Gao, Andrew McMinn, Min Wang

**Affiliations:** 1 MoE Key Laboratory of Evolution and Marine Biodiversity, College of Marine Life Sciences, Institute of Evolution and Marine Biodiversity, Key Lab of Polar Oceanography and Global Ocean Change, Frontiers Science Center for Deep Ocean, Center for Ocean Carbon Neutrality, Ocean University of China 12591, Qingdao, China; 2 Haide College, Ocean University of China 12591, Qingdao, China; 3 Institute for Marine and Antarctic Studies, University of Tasmania 3925, Hobart, Tasmania, Australia; 4 School of Biological Sciences, Victoria University of Wellington 8491, Wellington, New Zealand; 5 UMT-OUC Joint Centre for Marine Studies, Qingdao, China; Colorado School of Mines, Golden, Colorado, USA

**Keywords:** RNA virus, metatranscriptomic, Antarctic sea ice, viral diversity, phylogeny

## Abstract

Sea ice is vital to polar ecosystems and serves as a natural laboratory for studying microbial adaptations in extreme environments. Nevertheless, the diversity and ecology of Antarctic marine RNA viruses have not been sufficiently explored. Here, 11 metatranscriptomes from Antarctic sea ice and ice-associated environments were analyzed to characterize RNA viral communities and their ecological differentiation between sea ice and seawater. Diverse RNA viral lineages were identified, of which *Cryppavirales* and *Picornavirales* were dominant. *Lenarviricota* was predominant in sea ice, highlighting the differential distribution between the two ecosystems. Evolutionary analyses showed lower Ka/Ks values in sea ice-associated RNA viruses, suggesting stronger purifying constraints and a more conserved evolutionary pattern compared with under-ice seawater. More than half of the viral operational taxonomic units (vOTUs) could be assigned to hosts, primarily fungi and algae, suggesting a potential role for viruses in fungal–algal symbiotic/parasitic systems. This study provides new insights into the diversity and distinctiveness of RNA viruses in Antarctic sea ice–seawater systems.

**IMPORTANCE:**

Polar sea ice is a vital component of Antarctic ecosystems and plays an important role in climate regulation, yet the diversity and ecological roles of its viral communities remain largely unknown. This study documents the diversity and complexity of RNA viruses in Antarctic sea ice–seawater systems. The potential host lineages of RNA viruses in Antarctic sea ice were explored, shedding light on the cryptic RNA viral communities in these extreme environments. These findings provide important insights into the diversity and ecology of RNA viruses in these habitats, enhancing the understanding of polar viral ecology.

## INTRODUCTION

Sea ice is an important component of polar marine ecosystems. Prokaryotes and eukaryotes living in sea ice are important contributors to polar biogeochemical cycles, especially at the ice–seawater interface (1). Sea ice plays a key role in both the climate and ecology of the Southern Ocean (2). Climate changes, such as those caused by the greenhouse effect, have led to strong regional declines in sea ice extent (3). A new minimum summer Antarctic sea ice extent of 1.77 million square kilometers was reported on 19 February 2023, and this is 1.02 million square kilometers (36%) less than the average summer daily minimum from 1979 to 2022 (4, 5). The melting and thinning of sea ice are having impacts on polar microbial ecosystems and the survival of polar communities (6, 7).

Viruses are the most genetically diverse life forms in the ocean (8), and it is estimated that the global ocean contains more than 10^30^ virus-like particles. Viruses can infect all life forms from bacteria to whales (8, 9) and mediate horizontal gene transfer, in which DNA exchange can result in the acquisition of new functional genes that favor virus adaptation or help host metabolisms (10). Marine viruses are involved in the transition of billions of tons of organic matter every day. They are key drivers of host diversity, population dynamics, and biogeochemical cycling, and have important ecological functions (11). There is now growing evidence that RNA viruses, as well as DNA viruses, are abundant and ecologically important in marine ecosystems (12). Neri et al. explored the evolutionary relationships of RNA viruses globally, expanding the number of known RNA viruses by a factor of 5 in 2022 (13). Zayed et al. further explored the taxonomy and evolutionary origins of RNA viruses (14, 15). In addition, in contrast to DNA viruses, which mostly infect bacteria, RNA viruses tend to infect marine protists and other eukaryotes, mediating host communities and biogeochemistry (16, 17). However, the understanding of marine RNA viruses in extreme environments such as sea ice remains challenging due to difficult field conditions, access, and methodological inadequacies (18).

Previous studies have shown that viruses are abundant in sea ice (19), cryopeg brines (20), and other subzero brines (21). Some studies have also shown that the ratio of viruses to microorganisms in sea ice is higher than in seawater (22). Therefore, viruses likely play a key role in maintaining the stability of microbial communities in sea ice. The annual melting of the sea ice results in habitat switching between sea ice and seawater for microorganisms and viruses. However, a clear understanding of the dynamics of RNA viruses in sea ice and under-ice seawater is lacking.

Here, we analyzed five sea ice samples (ICE) and six under-ice seawater (SW) samples from McMurdo Sound in the Ross Sea, Antarctica, by using a metatranscriptomics approach. The structure of the RNA virus community and its potential hosts was investigated. This study contributes to the understanding of the diversity of RNA viral communities in and under sea ice and provides a baseline for further research on RNA viruses in extreme environments.

## RESULTS

### Antarctic sea ice database

**Fig 1:**
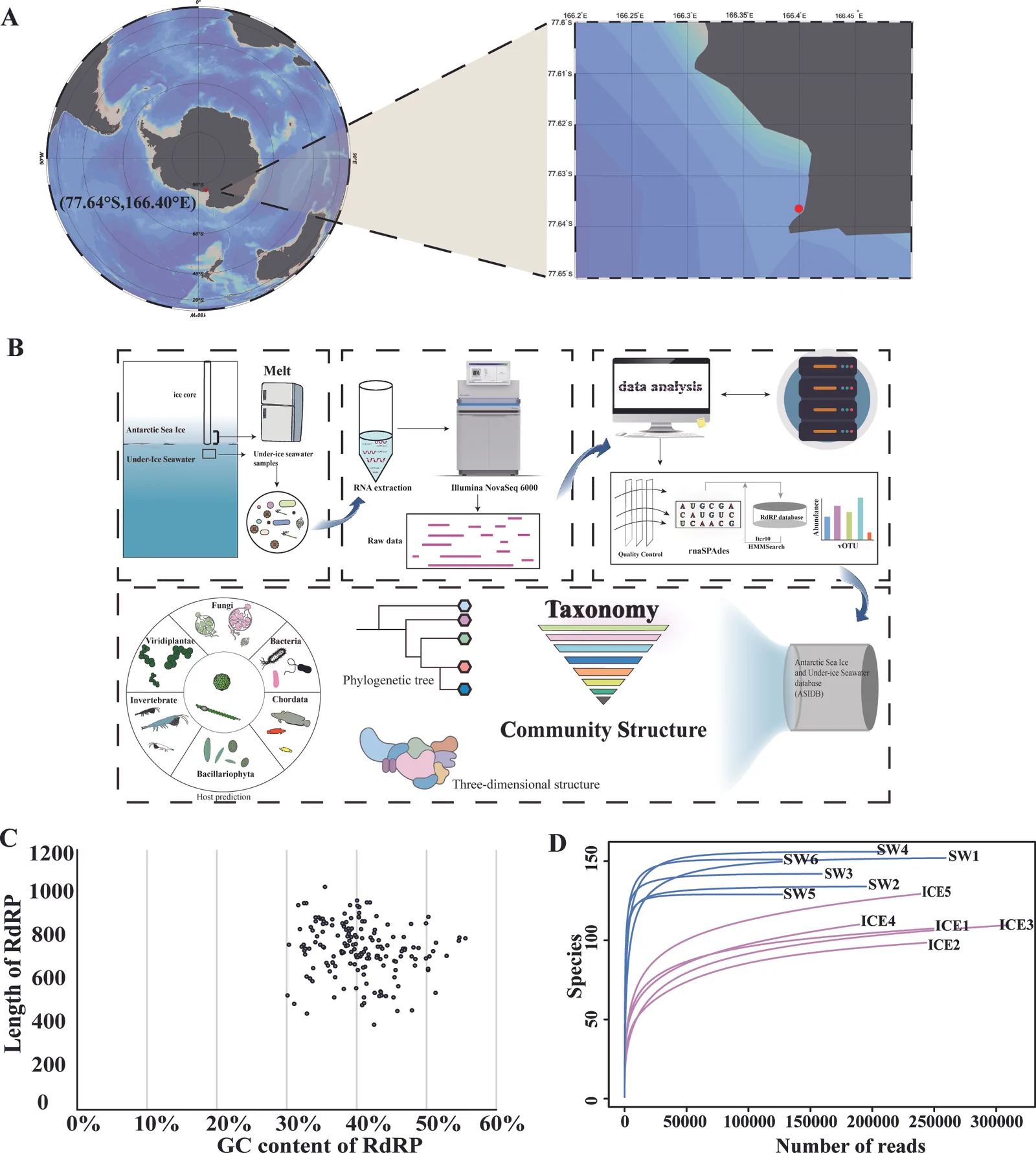
Antarctic sea ice database (ASIDB). (**A**) Geographic distribution of metatranscriptomics sampling sites involved in this study. The map of the sampling locations was created in Ocean Data View v5.6.3 (https://odv.awi.de/). A picture of the sample for this study is included as well. (**B**) Analyzing processes. (**C**) The length of RdRP and GC content identified in this study. (**D**) The rarefaction curves of each sample. ICE represents the sample of sea ice; SW represents the sample of under-ice seawater.

Eleven samples were obtained from the Cape Evans area of McMurdo Sound, Antarctica (Fig. 1A). Metatranscriptomics was used to investigate the diversity and ecological role of viruses inhabiting the ICE and SW samples (Fig. 1B). After assembly and trimming of raw reads, 1,203,458 contigs (>1 kb) were generated. Following quality control and RNA virus identification, 441 RNA viral contigs were detected, which were classified into 180 viral operational taxonomic units (vOTUs). ICE samples provided half of the vOTUs. The RNA-dependent RNA polymerase (RdRP) length of these vOTUs ranged from 400 to 1,000 bp, with most exhibiting GC contents between 30% and 50% (Fig. 1C). Rarefaction analysis indicated sufficient sampling coverage of viral diversity (Fig. 1D).

### Community structure of ICE and SW RNA viruses

**Fig 2:**
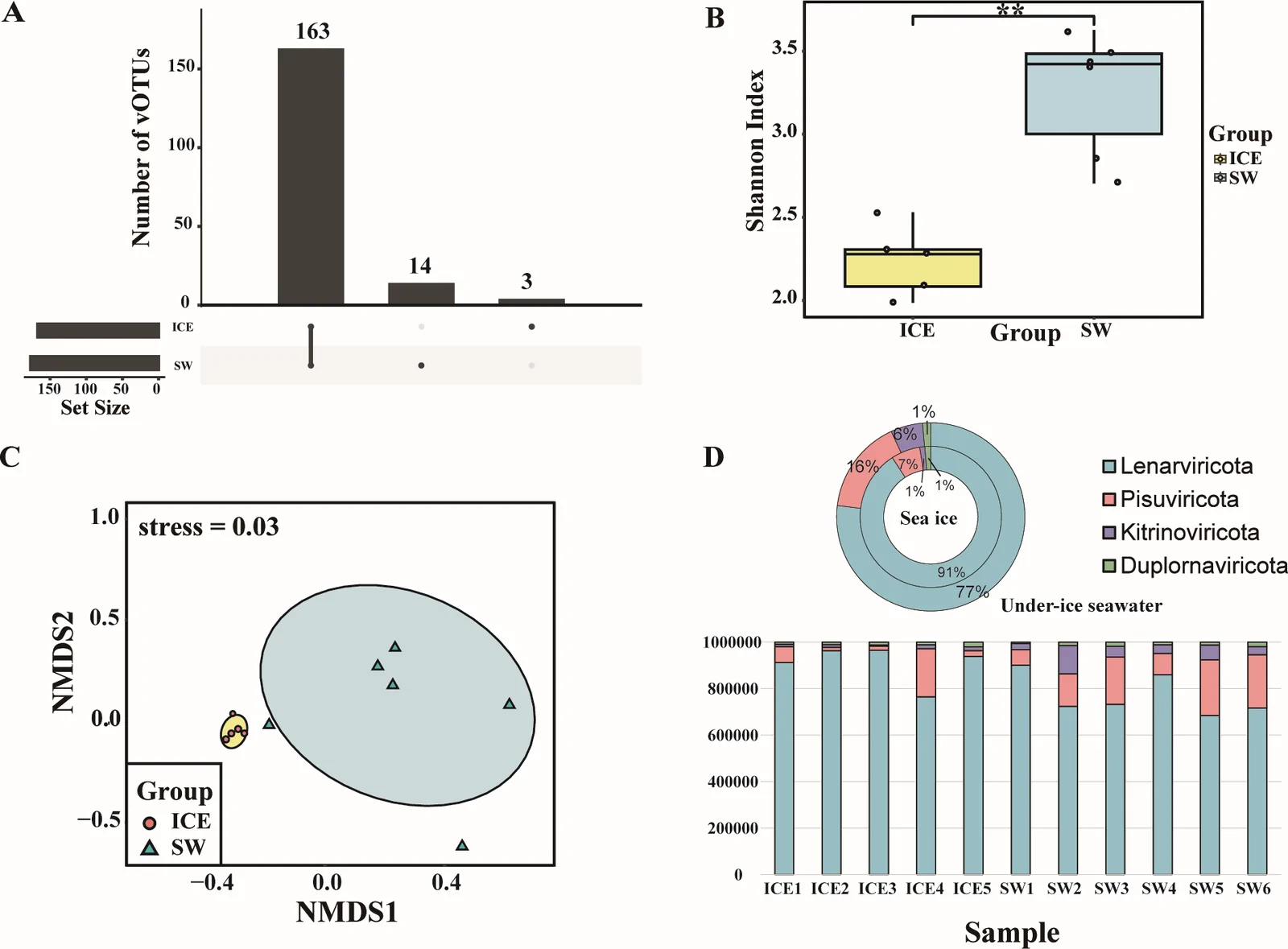
The diversity of RNA viruses in ICE and SW at both the vOTU level and the phylum level. (**A**) UpSet plots of shared vOTUs between ICE and SW. (**B**) Box plots show the median, upper, and lower quartile ranges and the variance among the samples. The difference between ICE and SW samples was determined with a Wilcoxon test, and significance was denoted by an asterisk (*P* < 0.01). (**C**) The non-metric multidimensional scaling (NMDS) plot of beta diversity illustrates the differences in viral community structure based on the relative abundance between ICE and SW samples. (**D**) Based on the relative abundance of viruses, the bar graphs show the number and classification (based on phylum level) of viral populations in each sample. Donut chart showing the distribution of RNA viruses in ICE and SW samples at the phylum level. ICE represents the sample of sea ice; SW represents the sample of under-ice seawater.

To examine the viral community structures of the ICE and SW samples, clean reads were mapped to vOTUs to calculate reads per kilobase per million mapped reads (RPKM) for each vOTU. Only 14 vOTUs were present in SW samples, 3 vOTUs were only in ICE samples (Fig. 2A), and these were classified into *Mitoviridae*, *Potyviridae,* and unclassified. For an assessment of community diversity, the Shannon–Wiener index of viral community structure was calculated. There was a significant difference (Wilcoxon test and *P* value less than 0.01) in the community diversity between ICE and SW samples, with the species richness of ICE communities being lower than that of SW (Fig. 2B; Fig. S1). The ICE group showed greater similarity in community composition than the SW group (Fig. 2C).

To better understand the composition of the RNA virus community, the vOTUs were annotated using the Viral Taxonomic Assignment Pipeline (VITAP) (23) and geNomad (24) and then blasted with the non-redundant protein sequences (nr) database. In the 11 samples, 4 viral phyla, 11 viral orders, and 13 viral families were annotated, with the majority belonging to *Lenarviricota* (accounting for 91% in ICE samples and 77% in SW samples) and *Pisuviricota* (accounting for 7% in ICE samples and 16% in SW samples) based on the abundance (Fig. 2D). Positive-sense single-stranded RNA (+ssRNA) viruses were the most abundant form in the RNA viromes. Based on RNA viral classification results, 12% of the viruses were not classified at the family level. Approximately 86% of the unclassified viruses could be categorized into two known viral phyla: *Pisuviricota* and *Duplornaviricota*.

To further explore how viral communities differ under selection pressure, the ratio of nonsynonymous to synonymous mutations was analyzed. An examination of the enrichment of RNA viruses in different conditions at the vOTUs level, using a *P* value of less than 0.05 in the *t*-test as the criterion, found six vOTUs were enriched in sea ice, 53 in under-ice seawater, and 121 with no significant difference between ICE and SW samples.

**Fig 3:**
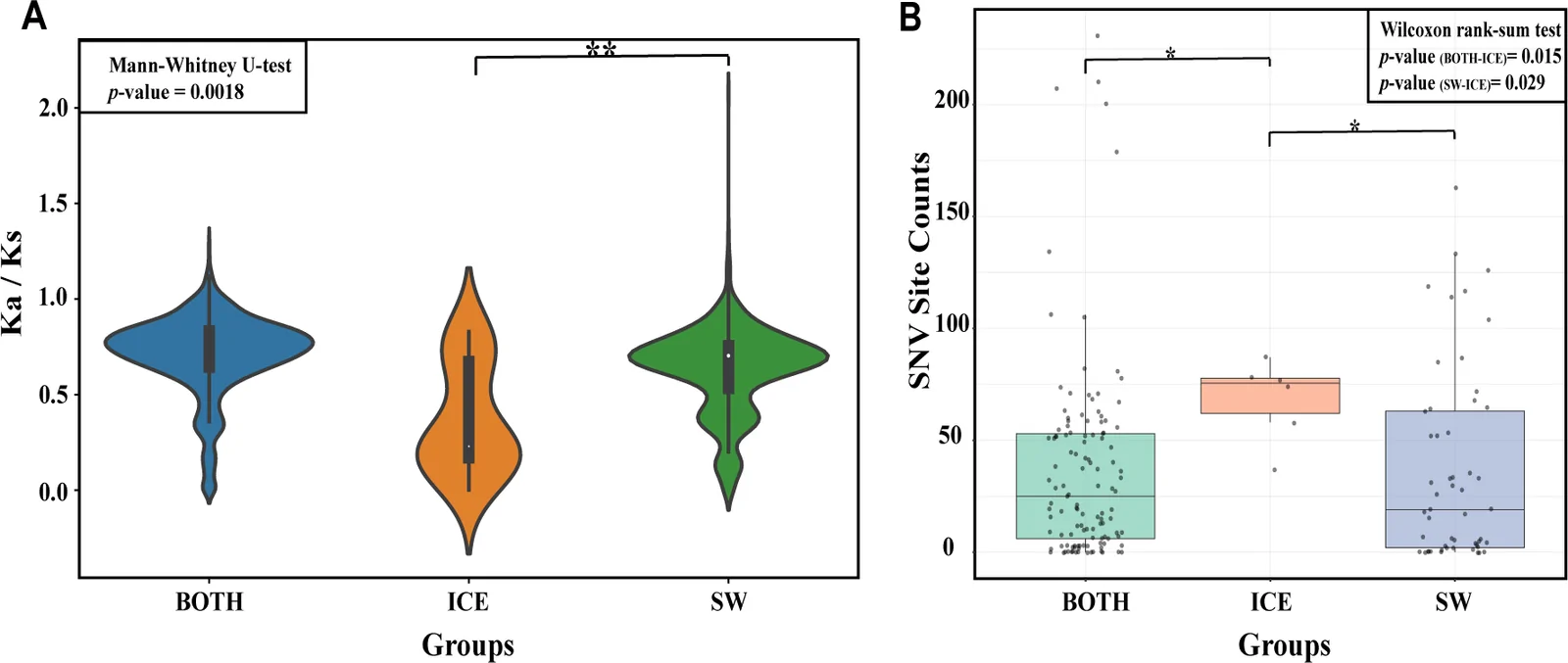
Selection pressure of RNA viruses enriched in ICE and SW samples. (**A**) Confidence intervals are plotted using a “violin” plot, including the median (white dot in the middle), 75% distribution (black bar), and 95% distribution (black line), with superimposed distribution (shaded area). ICE represents the viral sequences enriched in sea ice; SW represents the viral sequences enriched in under-ice seawater. BOTH represents the viral sequences with no significant enrichment. (**B**) The count of single-nucleotide variant sites in viral RdRP sequences was compared using Wilcoxon rank-sum tests.

Understanding the selection pressure on RNA viruses in different conditions allowed a better understanding of their adaptations in these two distinct environments. The selection pressure on RNA virus communities was assessed using the ratio of nonsynonymous to synonymous mutations (Ka/Ks) in the RdRP gene. The Ka/Ks ratios of RNA viruses differed among groups, with most values remaining below 1, indicating that RNA viruses in Antarctic sea ice and under-ice seawater were generally subject to purifying selection (Fig. 3A). Notably, ICE-associated RNA viruses showed significantly lower Ka/Ks ratios than SW-associated viruses (Mann–Whitney U test, *P* = 0.0018), suggesting stronger purifying constraints on viral protein-coding sequences in the sea ice environment (25). To further evaluate nucleotide-level variation, we calculated SNV site counts for RdRP sequences across the three groups (Fig. 3B). ICE-associated sequences showed relatively high SNV site counts, whereas BOTH and SW groups displayed broader distributions. The coexistence of relatively high SNV counts and low Ka/Ks ratios in ICE-associated viruses suggests that many nucleotide variants may be synonymous, low frequency, or selectively constrained, consistent with stronger purifying selection acting on RdRP protein sequences in sea ice.

### Divergent *Pisuviricota* lineages reveal underexplored RNA viral diversity in Antarctic sea ice

**Fig 4:**
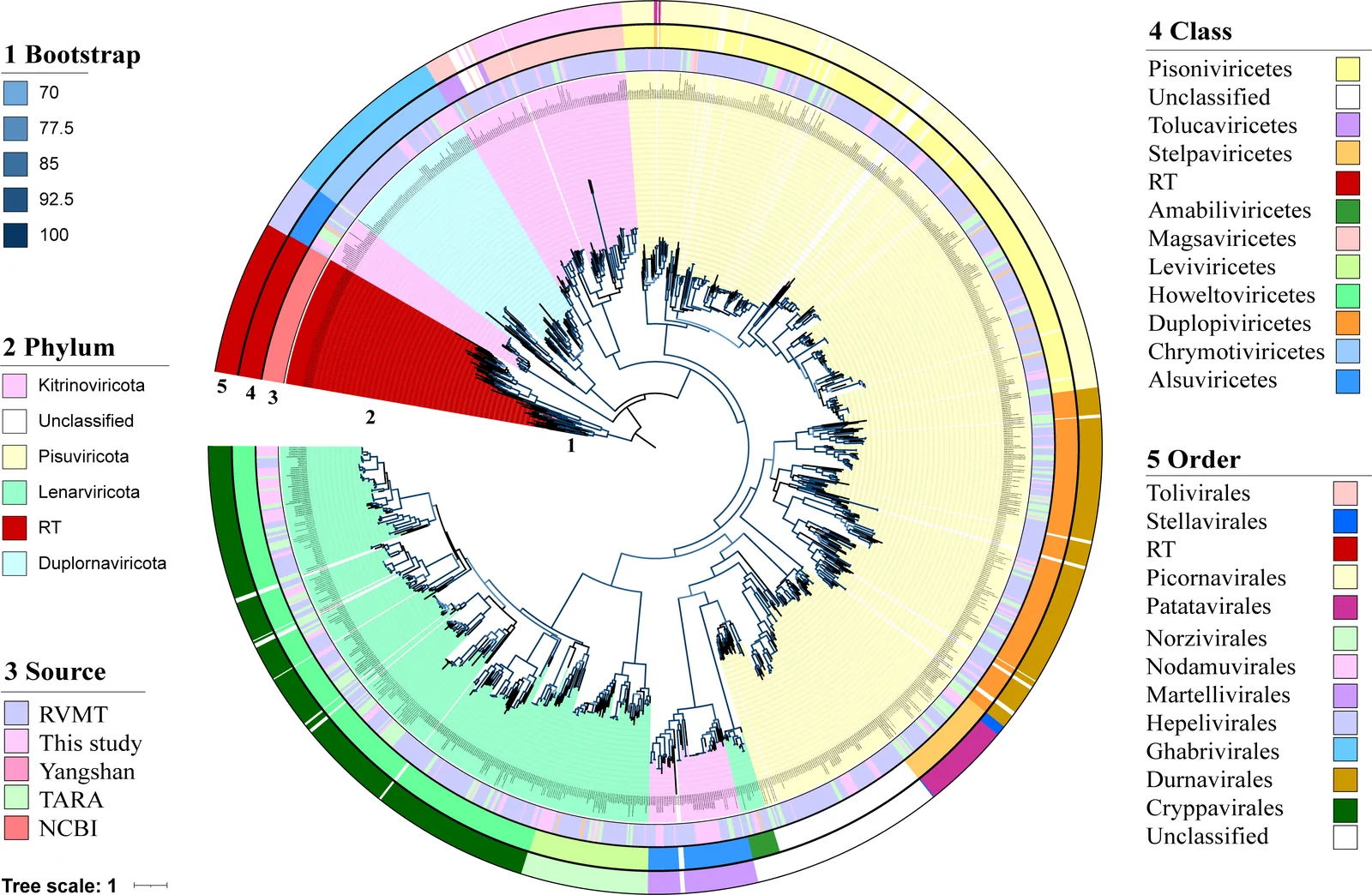
Phylogenetic analysis of RdRP. The phylogenetic tree was visualized using iTOL (https://itol.embl.de/) using reverse transcriptase as an outgroup and as the root of the maximum likelihood tree. The colors of phylogenetic tree branches represent bootstrap values. The inner ring (2) indicates the annotated phyla, the middle rings (3 and 4) indicate the source of the sequence and sequence-annotated class, and the outermost ring (5) indicates annotated order.

Although multiple annotation approaches were applied to improve taxonomic resolution, a subset of viral sequences could not be assigned to known viral taxa, indicating the presence of previously uncharacterized viruses in these environments. To refine the taxonomic assignment of RNA viruses based on marker genes, a phylogenetic analysis of RNA viruses using the RdRP marker gene was conducted. This approach allows for a more detailed classification and understanding of their evolutionary relationships (26, 27). Reverse transcriptase sequences were included as the outgroup (26). The phylogenetic tree was divided into five branches representing reverse transcriptase, *Lenarviricota*, *Kitrinoviricota*, *Pisuviricota,* and *Duplornavirico*ta. The phylogenetic tree of phylum *Pisuviricota* could be further divided into three subclades (viral orders): mainly including *Durnavirales*, *Picornavirales,* and *Patatavirales* (Fig. 4). Overall, the order-, class-, and family-level annotations were broadly consistent with the tree topology, supporting the reliability of the expanded taxonomic framework. Integrating the phylogenetic tree results, 68 vOTUs from this study were identified within *Pisuviricota*, with *Picornavirales* representing the most abundant order-level lineage. The expanded RdRP phylogeny resolved several major order-level lineages within *Pisuviricota*, including *Picornavirales*, *Durnavirales*, *Stellavirales, Ghabrivirales,* and *Patatavirales* (Fig. S2). Within this phylogenetic framework, *Patatavirales* formed an independent and well-defined cluster that was topologically positioned between the *Durnavirales*- and *Picornavirales*-associated clades. The close phylogenetic proximity among these three order-level groups indicates that *Patatavirales* may share a deeper evolutionary relationship with *Durnavirales* and *Picornavirales* while maintaining a separate lineage-level identity. Notably, a subset of sequences remained unclassified at the order, class, or family level despite the expanded reference comparison, suggesting that Antarctic sea ice and under-ice seawater harbor poorly characterized RNA viral lineages.

**Fig 5:**
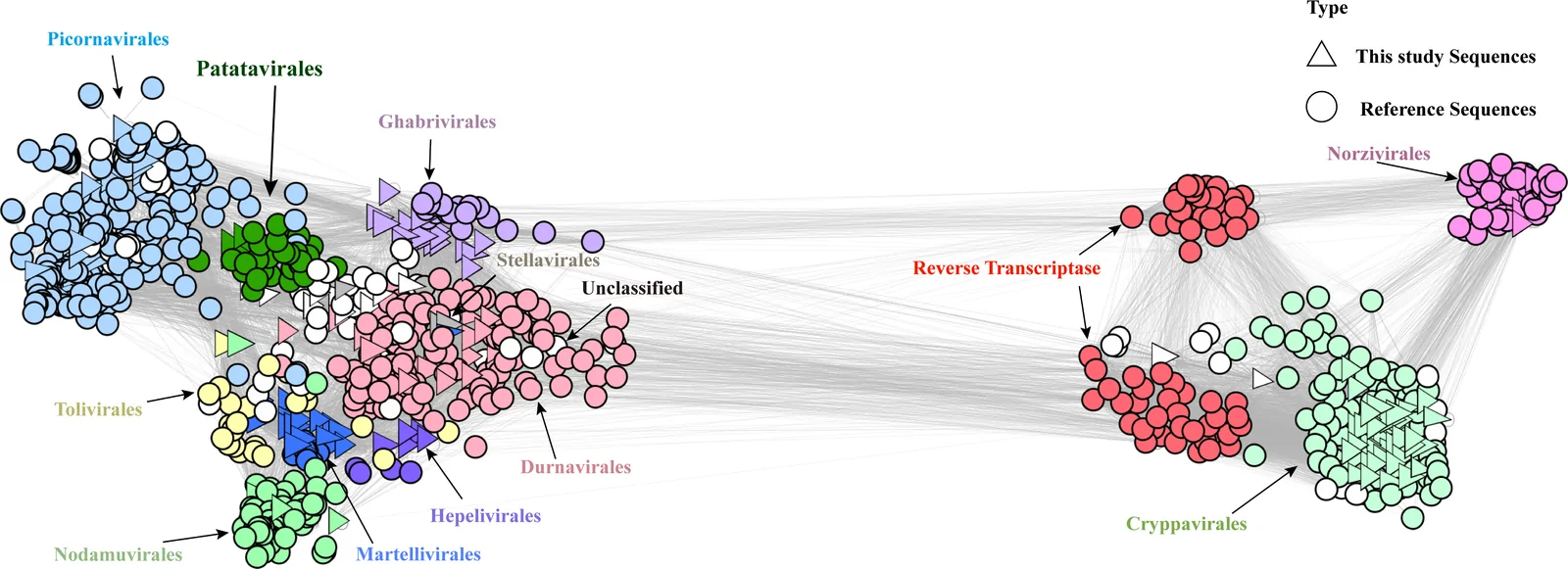
Three-dimensional structure similarity network of RdRP and reverse transcriptase protein domain. Each node represents a different structure. For clearer visualization, the top 100 best hits associated with each node are selected as significant nodes, with structure bit as the weight. Different colored nodes represent the commented virus order. The triangle represents the sequence from this study, and the circle represents the reference sequence.

The RdRP gene typically plays a major role in replication and transcription. The amino acid residues involved in certain key processes are often highly conserved (e.g., involved in catalysis and nucleotide selection) (28). Although RdRP proteins vary in domain architecture and sequence conservation among RNA viral lineages, their conserved structural features provide an additional framework for evaluating evolutionary relationships (29). To complement the RdRP phylogenetic analysis, three-dimensional (3D) structural similarity clustering was performed using RdRP sequences recovered in this study, reference RdRP sequences and reverse transcriptase sequences. The structural clustering analysis found that most major order-level groups formed recognizable structural clusters, including *Picornavirales*, *Durnavirales*, *Patatavirales*, *Cryppavirales*, and several other RNA viral lineages (Fig. 5). Reverse transcriptase sequences formed separate clusters from RNA viral RdRP sequences, supporting the distinction between the outgroup and RdRP structural groups. *Patatavirales* formed a compact structural cluster located close to the *Picornavirales* and *Durnavirales* clusters, consistent with its phylogenetic placement within the broader *Pisuviricota*-related RdRP diversity. Although some neighboring groups showed partial overlap in the structural similarity network, the overall clustering pattern was broadly consistent with the RdRP phylogeny and provided complementary evidence for the taxonomic placement of Antarctic RNA viral sequences. This pattern suggests that Antarctic sea ice and under-ice seawater have structurally diverse RNA viruses spanning multiple evolutionary lineages.

### RNA viruses in ICE and SW have diverse hosts

**Fig 6:**
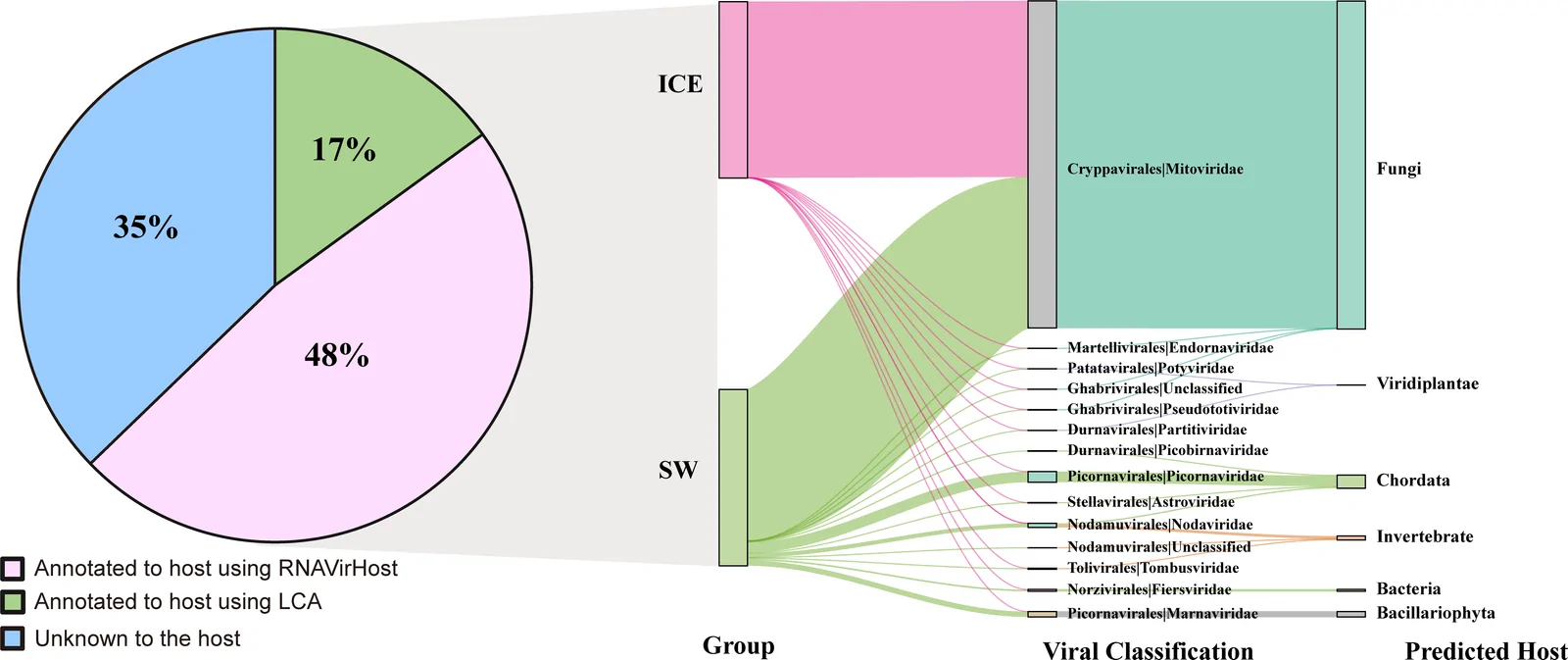
The connectivity between virus and potential hosts. The pie charts on the left represent the proportion of total vOTUs that are viruses predicted to connect to a host. Sankey diagram showing the relation among the sample group of RNA virus origin, the annotation of RNA viruses, and the predicted hypothetical host. ICE represents the samples of sea ice; SW represents the samples of under-ice seawater.

Viruses affect a large range of microbial-mediated processes through interactions with their hosts (30). Four methods were used to identify the hosts of RNA viruses in the ICE and SW samples. These included a comparison of RNA viruses with predicted bacteria and archaea CRISPR spacer sequences, available host information for viruses of established *Orthornaviran* taxa, RdRP protein sequence similarity to endogenous virus elements (EVEs), and RNAVirHost (31). Comparative analyses between CRISPR spacers and RNA viruses with their endogenous hosts yielded no significant alignment results. This may be primarily attributed to the inherent challenges associated with using CRISPR spacers to explore RNA viruses infecting prokaryotic organisms. Consequently, this study identified hosts based on a common lineage within the scope of virus families or at a more refined taxonomic level to which the viruses had been classified. Overall, 65% of the vOTUs could be linked to putative hosts, including 48% annotated using RNAVirHost and 17% inferred based on the last common ancestor (LCA) approach, whereas 35% remained without host assignment (Fig. 6). Consistent with current understanding, most known RNA viruses are associated with eukaryotic hosts (32). Host annotation revealed a dominance of eukaryotes, with most RNA viruses associated with fungi, chordata and photosynthetic eukaryotic lineages such as Bacillariophyta- and Viridiplantae-related groups (Fig. 6). Considering the marine context of this study, taxa currently assumed to infect Viridiplantae should instead be reinterpreted as infecting algae (33, 34).

*Cryppavirales* and *Picornavirales* were the dominant RNA viral lineages in both environments. At the family level, *Mitoviridae* within *Cryppavirales*, together with *Picornaviridae* and *Marnaviridae* within *Picornavirales*, represented the most abundant viral families (Fig. S2). Host prediction further showed that these dominant viral groups were mainly associated with eukaryotic host lineages. In particular, *Mitoviridae*-related vOTUs were primarily linked to fungi, whereas *Picornavirales*-related vOTUs were associated with diverse eukaryotic hosts, including Chordata and Bacillariophyta. Bacillariophyta, commonly known as diatoms, are major photosynthetic eukaryotes in marine and sea ice ecosystems. The prediction of Bacillariophyta-associated hosts suggests that some RNA viruses recovered in this study may be linked to diatom-related microbial lineages in Antarctic sea ice and under-ice seawater. This is consistent with the marine sea ice context of this study (35). These results suggest that the dominant RNA viral groups in Antarctic sea ice and under-ice seawater are associated with fungal and photosynthetic eukaryotic host lineages, providing a basis for further investigation of RNA virus–host interactions in polar microbial communities.

## DISCUSSION

Polar sea ice, which is one of the most extreme environments on Earth, harbors diverse microbial communities that are adapted to extreme cold and high salinity; collectively, they regulate the stability of the sea ice ecosystem (2, 36). However, with global warming, there has been a rapid reduction in sea ice extent (3, 37). Sea ice melting affects the community structure of the microorganisms, including viruses, living in the ice and the underlying seawater. Investigating the composition and diversity of RNA viral communities from the ICE and SW samples provides a basis for evaluating changes in sea ice ecosystems (38). Utilizing metatranscriptomic libraries, RNA viruses in Antarctic sea ice and under-ice seawater were investigated. In total, 441 viral contigs encoding the RdRPs belonging to 180 vOTUs were identified. In particular, the expansion of two viral orders, *Cryppavirales* and *Picornavirales*, accounted for 37% of the sequences from ICE and 56% from SW samples at vOTU level. This expands the known diversity of RNA viruses in sea ice ecosystems

The diversity of RNA viruses in ICE is considerably lower than that in SW (Fig. 2B), indicating that the drivers of community diversity in ICE and SW samples may be different at small scales. Sea ice microbial communities are derived from the underlying seawater during ice formation, but diversity is subsequently reduced by the selective pressure associated with the colder and more saline conditions; this may also influence viral survival and replication strategies (39, 40). RNA viruses enriched in ICE and SW were analyzed for selective pressure. It was found that the RNA viruses in ICE were under purifying selection, and the evolutionary trajectory was more conservative compared with the viruses from the SW samples (Fig. 3). It is worth speculating that environmental changes associated with freezing may have resulted in some of the RNA viruses not surviving, making the RNA virus community in ICE simpler than that in SW and reducing the abundance of RNA viruses in ICE relative to SW. This could be a contributing factor to the more conservative evolution of RNA viruses in ICE.

Sea ice algae are vital primary producers in the Antarctic ecosystem, providing essential food resources for predators such as copepods and krill (41). These organisms play a crucial role in supporting the marine food web, contributing significantly to the biological productivity and nutrient cycles in polar regions. Potential hosts for the RNA viruses in this study were examined using publicly available data on infected hosts from GenomeNet Database Resources. The results show that viruses with fungi, chordata, and photosynthetic eukaryotes as hosts were the most abundant. This re-interpretation is supported by two lines of evidence: the ecological predominance of algae in this environment (42) and their close phylogenetic relationship with plants. The sampling time of this study was during the Antarctic spring, when McMurdo Sound was in the early stages of a phytoplankton bloom (2, 43). This suggests that the high abundance of viruses is likely closely related to the abundance and diversity of their natural hosts. This is consistent with previous research, which indicates that RNA viruses may be particularly abundant and play a key ecological role during early-season phytoplankton blooms (44). The high abundance of RNA viruses with fungi and algae as their natural hosts in sea ice may further indicate that RNA viruses are closely associated with phytoplankton and interact with these potential hosts.

Polar sea ice is a key component of the Earth’s climate system and is essential for maintaining global climate stability and polar ecosystems (45). Viruses are integral members of the sea ice microbiome and the RNA, yet viral diversity in Antarctic sea ice has not previously been profiled. Based on metatranscriptomics, we systematically characterized the RNA viral community in Antarctic sea ice and under-ice seawater and evaluated selective pressure and potential hosts. Fungi and algae provided the majority of potential hosts for these RNA viruses. In addition, phylogenetic and structural similarity analyses found diverse and poorly characterized RNA viral lineages, indicating that Antarctic sea ice and ice-associated environments contain underexplored RNA viral diversity. The diversity of RNA viruses in sea ice was significantly lower than that in under-ice seawater, suggesting that habitat-associated environmental filtering and distinct evolutionary constraints may shape RNA viral community structure in these environments. This study provides new insights into the cryptic RNA viral communities of Antarctic sea ice and highlights their potential ecological relevance in polar microbial ecosystems.

## MATERIALS AND METHODS

### Sample collection, library construction, and RNA sequencing

Eleven samples for virus analysis were collected from Cape Evans, McMurdo Sound, Antarctica (77.64°S, 166.40°E), from 9 to 24 November 2019 (Fig. 1A); this included five sea ice samples and six under-ice seawater samples. Sea ice samples were collected using a SIPRE corer with the bottom 10 cm of ice retained. Sea ice samples were melted at 4℃. Seawater and melted sea ice samples were filtered onto 0.22 μm polycarbonate membrane filters after being pre-filtered through 3 μm filters. The filtered samples were then put in DNase/RNase-Free tubes, submerged in RNAlater reagent, and stored at −80°C. RNA viruses were extracted from the samples. Library construction and next-generation sequencing of RNA viruses were carried out using Illumina NovaSeq6000 (pair-end sequencing, 2 × 150 bp).

### Metatranscriptomic assembly and virus identification

The library adapters were removed, and raw reads were trimmed using Trimmomatic v0.39 (46) with default parameters. The rRNA was filtered using sortmerna (47) at the same time aligning the paired-end reads and output alignment reads into the FASTA/FASTQ file. The reads were assembled into contigs using rnaSPAdes (48) with default parameters.

Following the standard method of Uri Neri et al. (13), sequences with a contig length of less than 1,000 bp were discarded, as too short a sequence will lead to a significant increase in the mismatch rate. Prodigal software (49) was used to predict the open reading frame of contigs and select the meta procedure.

The RdRP gene is highly conserved among evolutionary distances of RNA viruses (29). Here, a homology search of the viral RdRP structural domain was performed using the hidden Markov model (HMM) approach HMMER v 3.1b2 (50) for the identification of viral contigs. Previously known sequences of RdRP proteins in the NCBI GenBank (version 233), sequences studied by Uri Neri et al. (13), global marine RNA sequences explored by Ahmed A. Zayed et al. (14), and analysis of an aquatic virome by Wolf et al. (51) were used to perform the iterative search.

With an alignment fraction greater than 70% as the threshold, the result of 10 iteration searches was performed, using MAFFT v7.475 (52) default parameters to obtain the best hits. To strictly reduce the presence of false positives, pfam_scan was used to annotate the iteratively searched proteins (53), specifying that the HMMER (54) bit score sequence cutoff for Pfam-A retrieval was 30 and preserving sequences with a length greater than 100 amino acids and a bit score greater than or equal to 30 as true positives for proteins containing the viral RdRP structural domains (14). Lower-scoring hits were manually checked for the presence of the previously reported RdRP structural domain motifs as previously reported (14, 28, 55). De-duplication was performed using BLASTN with a value of 10^−5^. Seqkit (56) is an efficient toolkit for handling fasta formatted files. Utilizing “seqkit translate,” the first filtered results were translated protein in six frames, and the HMM was used for iterative search to further accurately identify RNA virus RdRP.

Mmseqs (57) was used to calculate all pairwise sequence alignments in the contig set; for the output results, the average nucleotide identity (ANI) and alignment fraction (AF) values were calculated (see equations below [13]). When both ANI and AF values were greater than 90%, it was considered to be a vOTU. A comprehensive analysis was used to determine the vOTUs.


$$\begin{array}{l} ANI=\frac{\left(\%ID\times alignment\;length\right)}{Min(length\;of\;contigm.,\;length\;of\;contign.)}\\ AF=Min(alignment\;coverage\;of\;contigm.,\;alignment\;coverage\;of\;contign.) \end{array}$$

### Taxonomic annotation of RNA virus

To further understand the categories of RNA viruses, three methods were used: (i) VITAP with default parameters, (ii) geNomad (24) also with default parameters, and (iii) BLASTP (https://blast.ncbi.nlm.nih.gov/Blast.cgi) of RdRP sequences of this study to non-redundant protein sequence database (nr database) (https://www.ncbi.nlm.nih.gov/). Combining the three approaches provided an accurate understanding of the taxonomic information of viruses in the ICE and SW samples.

### Calculations of vOTU relative abundances and diversity

To enhance the reliability of the mapping, low-quality mapping results were eliminated. Based on a minimum read percentage of 0.95 and a minimum alignment occupancy of 0.75 threshold, using CoverM 0.6.1 (https://github.com/wwood/CoverM; with the command -m rpkm --min-read-percent-identity 0.95 --min-read-aligned-percent 0.75), the abundance of representative vOTUs with sequence lengths over 1,000 bp was calculated. All reads mapped to vOTUs were utilized to calculate the RPKM value. This process determined the coverage of each vOTU across various sample locations.

### Selection pressure

Based on the number of reads recovered by vOTU in the environment, sequence enrichment was assessed by performing a *t*-test, with a *P* value threshold of less than 0.05. This approach was used to determine the differential enrichment of sequences across environments and to generate the corresponding data sets. Protein sequence alignments were employed to guide the alignment of coding sequence (CDS), and the KaKs Calculator software (58) was utilized to compute the ratio of nonsynonymous (Ka) to synonymous (Ks) substitution rates for random binary combinations within each data set.

To quantify nucleotide-level variation in RdRP sequences, clean reads from each sample were mapped to the RdRP nucleotide sequences using Bowtie2 (59), and the resulting BAM files were sorted and indexed using SAMtools (60). Alignments were further filtered to retain reads with nucleotide identity ≥90%, calculated based on the NM tag and aligned length from the CIGAR string. Site-level nucleotide information was then extracted using SAMtools mpileup. Bases with quality scores lower than 20 were excluded.

### RdRP-based global marine phylogenetic tree

RdRP is frequently used as a signature gene for analyzing the deeply evolved development of RNA viruses (26). To further understand the taxonomy of RNA viruses in ICE and SW, a phylogenetic tree was constructed based on the representative RdRP gene. RdRP sequences from this study were compared with marine RNA virus sequences from databases including the RNA Viruses in Metatranscriptomes (RVMT) database, Yangshan database (51), and Tara Ocean Database (15) using DIAMOND Blastp (61), with an e-value threshold of less than 10^−3^ to obtain protein sequences relevant to this study. The RdRP sequences from this study, reference RdRP sequences and reverse transcriptases, were aligned using Muscle5 (62) with the -super5 option. Multiple sequence alignments were generated using MUSCLE5 by testing four guide-tree permutations and four perturbation seeds (0, 1, 2, and 3), and the best-scoring alignment was selected for downstream phylogenetic analysis. Only sequences containing less than 20% gaps were retained using trimAl (63). A phylogenetic tree was rooted with reverse transcriptase using IQ-TREE (64), with 1,000 bootstrap replicates. ModelFinder Plus was utilized to find the best-fit model for constructing the phylogenetic tree.

Following the overall phylogenetic tree analysis, separate phylogenetic trees were constructed for *Pisuviricota* to gain a more accurate understanding of their evolutionary relationships. The methodology for constructing phylum trees was consistent with that mentioned above.

### 3D structure network analysis

To investigate the three-dimensional structural similarity of RdRP domains within *Pisuviricota*, the most abundant phylum identified in this study, structural models of the corresponding RdRP proteins were predicted using ESMFold (65). Related reference sequences were identified from the reference data set described above using BLASTP with an E-value threshold of 1e^−5^. Pairwise structural comparisons of all RdRP three-dimensional models were then performed using Foldseek (66), with an e-value threshold of 10^−5^. Additionally, the three-dimensional structures of reverse transcriptases were incorporated as outgroups for subsequent analyses.

For each pair, the protein bitscore was utilized to construct a 3D structure network using the “Force Atlas” model in the Gephi software (67). Because the all-against-all structural comparison generated a large number of sequence pairs, each RdRP sequence recovered in this study and its top 100 most closely related reference sequences were selected for visualization.

### Inference of virus–host interactions

For the prediction of potential hosts, four approaches were adopted: (i) RNA virus sequences were compared to predicted bacteria and archaea CRISPR spacer sequences (13); (ii) available host information for viruses of established *Orthornaviran* taxa (14); (iii) RdRP protein sequence similarity to EVEs (68); and (ⅳ) a machine learning-based tool, RNAVirHost, was used to predict the hosts of RNA viruses (31).

Bacteria and archaea generate Clustered Regularly Interspaced Short Palindromic Repeats (CRISPRs) as part of their defense mechanism against phage invasions. During the assembly of these sequences, specific phage-derived sequence fragments, termed spacers, are integrated by the bacteria, enabling the rapid identification of invasive viruses. In this study, non-redundant RNA virus sequences were compared with predicted CRISPR spacers from bacteria and archaea in the IMG database (69) using BLASTn v2.11.0 with options ‘‘-dust no -word_size 7.” CRISPR arrays were selectively retained based on the following criteria: (i) only blastn hits with zero or one mismatch over the whole spacer length were preserved; (ii) the sequences matched required a minimum spacing of over 20 bp; (iii) the CRISPR array had to contain at least three spacers. Identified repeat sequences were then compared with bacterial and archaeal genomes in the IMG/M database using BLASTn (perc_identity 90 -dust no -word_size 7). Whether the host genomes associated with RNA virus spacers contained RT or Cas genes was also examined.

In the second approach, previously reported host information for established RNA viral taxa was used to infer putative hosts. vOTUs were first taxonomically annotated using geNomad, VITAP, and BLASTN. For vOTUs assigned at the family level or at a more resolved taxonomic rank, putative host lineages were inferred using a LCA-based strategy based on known hosts associated with the corresponding viral taxa. Virus–host information was obtained from the current virus–host database released by the International Committee on Taxonomy of Viruses (ICTV). Only viral taxa with available host information were included in this analysis.

The third method used RdRp protein sequence similarity to endogenous virus elements to search for hosts. The tblastn was performed between the RdRP of this study and the NCBI sequence database, including bacteria, archaea, fungi, invertebrates, plants, protists, and vertebrates (1 × 10^−20^ for e-value).

The fourth method used integrating genomic traits and sequence homologies to predict the likely host of infection through RNAVirHOST.

### Statistical analysis

R version 4.3.2, ggplot2 (v3.4.4), and vegan (v2.6-4) packages were used to calculate the Shannon–Wiener index, perform Wilcoxon tests for inter-sample differences, and conduct alpha diversity and NMDS analysis (70, 71). These packages were also used to generate rarefaction curves and scatter plots, facilitating the visualization of the data. Violin plots were generated using Python, leveraging the seaborn and matplotlib.pyplot libraries for data visualization. Differences in SNV site counts among groups were evaluated using pairwise Wilcoxon rank-sum tests with Benjamini–Hochberg correction. The “Force Atlas” model in the Gephi program was used to create a 3D structural network utilizing the protein bitscore. The phylogenetic tree was visualized using iTOL (72). Visualization of the relationship between viruses and potential hosts used the online website (https://sankeymatic.com/build/).

## Data Availability

The raw read data reported in this paper have been deposited in the Genome Sequence Archive (73) in the National Genomics Data Center (74), China National Center for Bioinformation/Beijing Institute of Genomics, Chinese Academy of Sciences (GSA: CRA020436).

## SUPPLEMENTAL MATERIAL

Supplemental material aem.01472-26-s0001.pdf Fig. S1 and S2.

ASM does not own the copyrights to Supplemental Material that may be linked to, or accessed through, an article. The authors have granted ASM a non-exclusive, world-wide license to publish the Supplemental Material files. Please contact the corresponding author directly for reuse.

## References

[1] Vancoppenolle M, Meiners KM, Michel C, Bopp L, Brabant F, Carnat G, Delille B, Lannuzel D, Madec G, Moreau S, Tison J-L, van der Merwe P. 2013. Role of sea ice in global biogeochemical cycles: emerging views and challenges. Quat Sci Rev 79:207–230. doi:10.1016/j.quascirev.2013.04.011

[2] Boetius A, Anesio AM, Deming JW, Mikucki JA, Rapp JZ. 2015. Microbial ecology of the cryosphere: sea ice and glacial habitats. Nat Rev Microbiol 13:677–690. doi:10.1038/nrmicro3522 26344407

[3] Lauber J, Hattermann T, de Steur L, Darelius E, Auger M, Nøst OA, Moholdt G. 2023. Warming beneath an East Antarctic ice shelf due to increased subpolar westerlies and reduced sea ice. Nat Geosci 16:877–885. doi:10.1038/s41561-023-01273-5

[4] Purich A, Doddridge EW. 2023. Record low Antarctic sea ice coverage indicates a new sea ice state. Commun Earth Environ 4:1–9. doi:10.1038/s43247-023-00961-9

[5] Fetterer F, Knowles K, Knowles K, Savoie M, Windnagel AK. 2021. Sea Ice Index. (G02135, Version 3). NSIDC: National Snow and Ice Data Center. 10.7265/N5K072F8.

[6] Wu B, Li Z. 2022. Possible impacts of anomalous Arctic sea ice melting on summer atmosphere. Intl Journal of Climatology 42:1818–1827. doi:10.1002/joc.7337

[7] Boras JA, Sala MM, Arrieta JM, Sà EL, Felipe J, Agustí S, Duarte CM, Vaqué D. 2010. Effect of ice melting on bacterial carbon fluxes channelled by viruses and protists in the Arctic Ocean. Polar Biol 33:1695–1707. doi:10.1007/s00300-010-0798-8

[8] Suttle CA. 2005. Viruses in the sea. Nature 437:356–361. doi:10.1038/nature04160 16163346

[9] Brussaard CPD, Wilhelm SW, Thingstad F, Weinbauer MG, Bratbak G, Heldal M, Kimmance SA, Middelboe M, Nagasaki K, Paul JH, Schroeder DC, Suttle CA, Vaqué D, Wommack KE. 2008. Global-scale processes with a nanoscale drive: the role of marine viruses. ISME J 2:575–578. doi:10.1038/ismej.2008.31 18385772

[10] Hurwitz BL, Hallam SJ, Sullivan MB. 2013. Metabolic reprogramming by viruses in the sunlit and dark ocean. Genome Biol 14:R123. doi:10.1186/gb-2013-14-11-r123 PMC4053976 24200126

[11] Coutinho FH, Silveira CB, Gregoracci GB, Thompson CC, Edwards RA, Brussaard CPD, Dutilh BE, Thompson FL. 2017. Marine viruses discovered via metagenomics shed light on viral strategies throughout the oceans. Nat Commun 8:15955. doi:10.1038/ncomms15955 PMC5504273 28677677

[12] Steward GF, Culley AI, Mueller JA, Wood-Charlson EM, Belcaid M, Poisson G. 2013. Are we missing half of the viruses in the ocean? ISME J 7:672–679. doi:10.1038/ismej.2012.121 PMC3578568 23151645

[13] Neri U, Wolf YI, Roux S, Camargo AP, Lee B, Kazlauskas D, Chen IM, Ivanova N, Zeigler Allen L, Paez-Espino D, Bryant DA, Bhaya D, Krupovic M, Dolja VV, Kyrpides NC, Koonin EV, Gophna U, RNA Virus Discovery Consortium. 2022. Expansion of the global RNA virome reveals diverse clades of bacteriophages. Cell 185:4023–4037. doi:10.1016/j.cell.2022.08.023 36174579

[14] Zayed AA, Wainaina JM, Dominguez-Huerta G, Pelletier E, Guo J, Mohssen M, Tian F, Pratama AA, Bolduc B, Zablocki O, et al.. 2022. Cryptic and abundant marine viruses at the evolutionary origins of Earth’s RNA virome. Science 376:156–162. doi:10.1126/science.abm5847 PMC10990476 35389782

[15] Dominguez-Huerta G, Zayed AA, Wainaina JM, Guo J, Tian F, Pratama AA, Bolduc B, Mohssen M, Zablocki O, Pelletier E, et al.. 2022. Diversity and ecological footprint of Global Ocean RNA viruses. Science 376:1202–1208. doi:10.1126/science.abn6358 35679415

[16] Coy SR, Utama B, Spurlin JW, Kim JG, Deshmukh H, Lwigale P, Nagasaki K, Correa AMS. 2023. Visualization of RNA virus infection in a marine protist with a universal biomarker. Sci Rep 13:5813. doi:10.1038/s41598-023-31507-w PMC10086069 37037845

[17] Zeigler Allen L, McCrow JP, Ininbergs K, Dupont CL, Badger JH, Hoffman JM, Ekman M, Allen AE, Bergman B, Venter JC. 2017. The baltic sea virome: diversity and transcriptional activity of DNA and RNA viruses. mSystems 2:e00125-16. doi:10.1128/mSystems.00125-16 PMC5309335 28217745

[18] Culley A. 2018. New insight into the RNA aquatic virosphere via viromics. Virus Res 244:84–89. doi:10.1016/j.virusres.2017.11.008 29138044

[19] Wells LE, Deming JW. 2006. Modelled and measured dynamics of viruses in Arctic winter sea-ice brines. Environ Microbiol 8:1115–1121. doi:10.1111/j.1462-2920.2006.00984.x 16689732

[20] Zhong Z-P, Rapp JZ, Wainaina JM, Solonenko NE, Maughan H, Carpenter SD, Cooper ZS, Jang HB, Bolduc B, Deming JW, Sullivan MB. 2020. Viral ecogenomics of arctic cryopeg brine and sea ice. mSystems 5:e00246-20. doi:10.1128/mSystems.00246-20 PMC7300359 32546670

[21] Colangelo-Lillis J, Eicken H, Carpenter SD, Deming JW. 2016. Evidence for marine origin and microbial-viral habitability of sub-zero hypersaline aqueous inclusions within permafrost near Barrow, Alaska. FEMS Microbiol Ecol 92:fiw053. doi:10.1093/femsec/fiw053 26976841

[22] Collins RE, Deming JW. 2011. Abundant dissolved genetic material in Arctic sea ice Part II: Viral dynamics during autumn freeze-up. Polar Biol 34:1831–1841. doi:10.1007/s00300-011-1008-z

[23] Zheng K, Sun J, Liang Y, Kong L, Paez-Espino D, Mcminn A, Wang M. 2025. VITAP: a high precision tool for DNA and RNA viral classification based on meta-omic data. Nat Commun 16. doi:10.1038/s41467-025-57500-7 PMC11883027 40044690

[24] Camargo AP, Roux S, Schulz F, Babinski M, Xu Y, Hu B, Chain PSG, Nayfach S, Kyrpides NC. 2024. Identification of mobile genetic elements with geNomad. Nat Biotechnol 42:1303–1312. doi:10.1038/s41587-023-01953-y PMC11324519 37735266

[25] Hurst LD. 2002. The Ka/Ks ratio: diagnosing the form of sequence evolution. Trends in Genetics 18:486–487. doi:10.1016/S0168-9525(02)02722-1 12175810

[26] Wolf YI, Kazlauskas D, Iranzo J, Lucía-Sanz A, Kuhn JH, Krupovic M, Dolja VV, Koonin EV. 2018. Origins and evolution of the global RNA virome. mBio 9:e02329-18. doi:10.1128/mBio.02329-18 PMC6282212 30482837

[27] Hillary LS, Adriaenssens EM, Jones DL, McDonald JE. 2022. RNA-viromics reveals diverse communities of soil RNA viruses with the potential to affect grassland ecosystems across multiple trophic levels. ISME Commun 2:34. doi:10.1038/s43705-022-00110-x PMC8992426 36373138

[28] Bruenn JA. 2003. A structural and primary sequence comparison of the viral RNA-dependent RNA polymerases. Nucleic Acids Res 31:1821–1829. doi:10.1093/nar/gkg277 PMC152793 12654997

[29] Iyer LM, Koonin EV, Aravind L. 2003. Evolutionary connection between the catalytic subunits of DNA-dependent RNA polymerases and eukaryotic RNA-dependent RNA polymerases and the origin of RNA polymerases. BMC Struct Biol 3:1. doi:10.1186/1472-6807-3-1 PMC151600 12553882

[30] Breitbart M. 2012. Marine viruses: truth or dare. Annu Rev Mar Sci 4:425–448. doi:10.1146/annurev-marine-120709-142805 22457982

[31] Chen G, Jiang J, Sun Y. 2024. RNAVirHost: a machine learning-based method for predicting hosts of RNA viruses through viral genomes. Gigascience 13:giae059. doi:10.1093/gigascience/giae059 PMC11340644 39172545

[32] Sadeghi M, Tomaru Y, Ahola T. 2021. RNA viruses in aquatic unicellular eukaryotes. Viruses 13:362. doi:10.3390/v13030362 PMC7996518 33668994

[33] Hess S, Williams SK, Busch A, Irisarri I, Delwiche CF, de Vries S, Darienko T, Roger AJ, Archibald JM, Buschmann H, von Schwartzenberg K, de Vries J. 2022. A phylogenomically informed five-order system for the closest relatives of land plants. Curr Biol 32:4473–4482. doi:10.1016/j.cub.2022.08.022 PMC9632326 36055238

[34] Chandola U, Manirakiza E, Maillard M, Lavier Aydat LJ, Camuel A, Trottier C, Tanaka A, Chaumier T, Giraud E, Tirichine L. 2025. A Bradyrhizobium isolate from a marine diatom induces nitrogen-fixing nodules in a terrestrial legume. Nat Microbiol 10:2486–2497. doi:10.1038/s41564-025-02105-5 40913088

[35] Lang AS, Vlok M, Culley AI, Suttle CA, Takao Y, Tomaru Y, ICTV Report Consortium. 2021. ICTV virus taxonomy profile: marnaviridae 2021. J Gen Virol 102:001633. doi:10.1099/jgv.0.001633 PMC8513639 34356002

[36] Priscu JC, Laybourn-Parry J, Häggblom M. 2014. Polar and alpine microbiology in a changing world. FEMS Microbiol Ecol 89:209–210. doi:10.1111/1574-6941.12371 25092542

[37] Roach LA, Meier WN. 2024. Sea ice in 2023. Nat Rev Earth Environ 5:235–237. doi:10.1038/s43017-024-00542-0

[38] Cavicchioli R, Ripple WJ, Timmis KN, Azam F, Bakken LR, Baylis M, Behrenfeld MJ, Boetius A, Boyd PW, Classen AT, et al.. 2019. Scientists’ warning to humanity: microorganisms and climate change. Nat Rev Microbiol 17:569–586. doi:10.1038/s41579-019-0222-5 PMC7136171 31213707

[39] Atanasova NS, Bamford DH, Oksanen HM. 2016. Virus-host interplay in high salt environments. Environ Microbiol Rep 8:431–444. doi:10.1111/1758-2229.12385 26929102

[40] Gregory AC, Zayed AA, Conceição-Neto N, Temperton B, Bolduc B, Alberti A, Ardyna M, Arkhipova K, Carmichael M, Cruaud C, et al.. 2019. Marine DNA viral macro- and microdiversity from pole to pole. Cell 177:1109–1123. doi:10.1016/j.cell.2019.03.040 PMC6525058 31031001

[41] Zhang Z, Qu C, Zhang K, He Y, Zhao X, Yang L, Zheng Z, Ma X, Wang X, Wang W, Wang K, Li D, Zhang L, Zhang X, Su D, Chang X, Zhou M, Gao D, Jiang W, Leliaert F, Bhattacharya D, De Clerck O, Zhong B, Miao J. 2020. Adaptation to extreme Antarctic environments revealed by the genome of a sea ice green alga. Curr Biol 30:3330–3341. doi:10.1016/j.cub.2020.06.029 32619486

[42] Lasbleiz M, Leblanc K, Armand LK, Christaki U, Georges C, Obernosterer I, Quéguiner B. 2016. Composition of diatom communities and their contribution to plankton biomass in the naturally iron-fertilized region of Kerguelen in the Southern Ocean. FEMS Microbiol Ecol 92:fiw171. doi:10.1093/femsec/fiw171 27515734

[43] Ducklow HW, Baker K, Martinson DG, Quetin LB, Ross RM, Smith RC, Stammerjohn SE, Vernet M, Fraser W. 2007. Marine pelagic ecosystems: the west Antarctic Peninsula. Philos Trans R Soc Lond B Biol Sci 362:67–94. doi:10.1098/rstb.2006.1955 PMC1764834 17405208

[44] Miranda JA, Culley AI, Schvarcz CR, Steward GF. 2016. RNA viruses as major contributors to Antarctic virioplankton. Environ Microbiol 18:3714–3727. doi:10.1111/1462-2920.13291 26950773

[45] Post E, Bhatt US, Bitz CM, Brodie JF, Fulton TL, Hebblewhite M, Kerby J, Kutz SJ, Stirling I, Walker DA. 2013. Ecological consequences of sea-ice decline. Science 341:519–524. doi:10.1126/science.1235225 23908231

[46] Bolger AM, Lohse M, Usadel B. 2014. Trimmomatic: a flexible trimmer for Illumina sequence data. Bioinformatics 30:2114–2120. doi:10.1093/bioinformatics/btu170 PMC4103590 24695404

[47] Kopylova E, Noé L, Touzet H. 2012. SortMeRNA: fast and accurate filtering of ribosomal RNAs in metatranscriptomic data. Bioinformatics 28:3211–3217. doi:10.1093/bioinformatics/bts611 23071270

[48] Bushmanova E, Antipov D, Lapidus A, Prjibelski AD. 2019. rnaSPAdes: a *de novo* transcriptome assembler and its application to RNA-Seq data. Gigascience 8:giz100. doi:10.1093/gigascience/giz100 PMC6736328 31494669

[49] Hyatt D, Chen G-L, Locascio PF, Land ML, Larimer FW, Hauser LJ. 2010. Prodigal: prokaryotic gene recognition and translation initiation site identification. BMC Bioinformatics 11:119. doi:10.1186/1471-2105-11-119 PMC2848648 20211023

[50] Grewal JK, Krzywinski M, Altman N. 2019. Markov models — hidden Markov models. Nat Methods 16:795–796. doi:10.1038/s41592-019-0532-6 31471599

[51] Wolf YI, Silas S, Wang Y, Wu S, Bocek M, Kazlauskas D, Krupovic M, Fire A, Dolja VV, Koonin EV. 2020. Doubling of the known set of RNA viruses by metagenomic analysis of an aquatic virome. Nat Microbiol 5:1262–1270. doi:10.1038/s41564-020-0755-4 PMC7508674 32690954

[52] Rozewicki J, Li S, Amada KM, Standley DM, Katoh K. 2019. MAFFT-DASH: integrated protein sequence and structural alignment. Nucleic Acids Res 47:W5–W10. doi:10.1093/nar/gkz342 PMC6602451 31062021

[53] Mistry J, Chuguransky S, Williams L, Qureshi M, Salazar GA, Sonnhammer ELL, Tosatto SCE, Paladin L, Raj S, Richardson LJ, Finn RD, Bateman A. 2021. Pfam: the protein families database in 2021. Nucleic Acids Res 49:D412–D419. doi:10.1093/nar/gkaa913 PMC7779014 33125078

[54] Eddy SR. 2011. Accelerated profile HMM searches. PLoS Comput Biol 7:e1002195. doi:10.1371/journal.pcbi.1002195 PMC3197634 22039361

[55] te Velthuis AJW. 2014. Common and unique features of viral RNA-dependent polymerases. Cell Mol Life Sci 71:4403–4420. doi:10.1007/s00018-014-1695-z PMC4207942 25080879

[56] Shen W, Le S, Li Y, Hu F. 2016. SeqKit: a cross-platform and ultrafast toolkit for FASTA/Q file manipulation. PLoS One 11:e0163962. doi:10.1371/journal.pone.0163962 PMC5051824 27706213

[57] Steinegger M, Söding J. 2017. MMseqs2 enables sensitive protein sequence searching for the analysis of massive data sets. Nat Biotechnol 35:1026–1028. doi:10.1038/nbt.3988 29035372

[58] Zhang Z, Li J, Zhao X-Q, Wang J, Wong GK-S, Yu J. 2006. KaKs_calculator: calculating Ka and Ks through model selection and model averaging. Genomics Proteomics Bioinformatics 4:259–263. doi:10.1016/S1672-0229(07)60007-2 PMC5054075 17531802

[59] Langmead B, Salzberg SL. 2012. Fast gapped-read alignment with Bowtie 2. Nat Methods 9:357–359. doi:10.1038/nmeth.1923 PMC3322381 22388286

[60] Danecek P, Bonfield JK, Liddle J, Marshall J, Ohan V, Pollard MO, Whitwham A, Keane T, McCarthy SA, Davies RM, Li H. 2021. Twelve years of SAMtools and BCFtools. Gigascience 10:giab008. doi:10.1093/gigascience/giab008 PMC7931819 33590861

[61] Buchfink B, Xie C, Huson DH. 2015. Fast and sensitive protein alignment using DIAMOND. Nat Methods 12:59–60. doi:10.1038/nmeth.3176 25402007

[62] Edgar RC. 2022. Muscle5: high-accuracy alignment ensembles enable unbiased assessments of sequence homology and phylogeny. Nat Commun 13:6968. doi:10.1038/s41467-022-34630-w PMC9664440 36379955

[63] Capella-Gutiérrez S, Silla-Martínez JM, Gabaldón T. 2009. trimAl: a tool for automated alignment trimming in large-scale phylogenetic analyses. Bioinformatics 25:1972–1973. doi:10.1093/bioinformatics/btp348 PMC2712344 19505945

[64] Nguyen L-T, Schmidt HA, von Haeseler A, Minh BQ. 2015. IQ-TREE: a fast and effective stochastic algorithm for estimating maximum-likelihood phylogenies. Mol Biol Evol 32:268–274. doi:10.1093/molbev/msu300 PMC4271533 25371430

[65] Lin Z, Akin H, Rao R, Hie B, Zhu Z, Lu W, Smetanin N, Verkuil R, Kabeli O, Shmueli Y, Dos Santos Costa A, Fazel-Zarandi M, Sercu T, Candido S, Rives A. 2023. Evolutionary-scale prediction of atomic-level protein structure with a language model. Science 379:1123–1130. doi:10.1126/science.ade2574 36927031

[66] van Kempen M, Kim SS, Tumescheit C, Mirdita M, Lee J, Gilchrist CLM, Söding J, Steinegger M. 2024. Fast and accurate protein structure search with Foldseek. Nat Biotechnol 42:243–246. doi:10.1038/s41587-023-01773-0 PMC10869269 37156916

[67] Bastian M, Heymann S, Jacomy M. 2009. Gephi: an open source software for exploring and manipulating networks. ICWSM 3:361–362. doi:10.1609/icwsm.v3i1.13937

[68] Shi M, Lin X-D, Tian J-H, Chen L-J, Chen X, Li C-X, Qin X-C, Li J, Cao J-P, Eden J-S, Buchmann J, Wang W, Xu J, Holmes EC, Zhang Y-Z. 2016. Redefining the invertebrate RNA virosphere. Nature 540:539–543. doi:10.1038/nature20167 27880757

[69] Mukherjee S, Stamatis D, Li CT, Ovchinnikova G, Bertsch J, Sundaramurthi JC, Kandimalla M, Nicolopoulos PA, Favognano A, Chen I-M, Kyrpides NC, Reddy TBK. 2023. Twenty-five years of genomes OnLine database (GOLD): data updates and new features in v.9. Nucleic Acids Res 51:D957–D963. doi:10.1093/nar/gkac974 PMC9825498 36318257

[70] Dixon P. 2003. VEGAN, a package of R functions for community ecology. J Vegetation Science 14:927–930. doi:10.1111/j.1654-1103.2003.tb02228.x

[71] Wickham H. 2009. ggplot2: elegant graphics for data analysis. Springer, New York.

[72] Letunic I, Bork P. 2024. Interactive Tree of Life (iTOL) v6: recent updates to the phylogenetic tree display and annotation tool. Nucleic Acids Res 52:W78–W82. doi:10.1093/nar/gkae268 PMC11223838 38613393

[73] Chen T, Chen X, Zhang S, Zhu J, Tang B, Wang A, Dong L, Zhang Z, Yu C, Sun Y, Chi L, Chen H, Zhai S, Sun Y, Lan L, Zhang X, Xiao J, Bao Y, Wang Y, Zhang Z, Zhao W. 2021. The genome sequence archive family: toward explosive data growth and diverse data types. Genomics Proteomics Bioinformatics 19:578–583. doi:10.1016/j.gpb.2021.08.001 PMC9039563 34400360

[74] Xue Y, Bao Y, Zhang Z, Zhao W, Xiao J, He S, Zhang G, Li Y, Zhao G, Chen R, et al.. 2022. Database resources of the national genomics data center, China national center for bioinformation in 2022. Nucleic Acids Res 50:D27–D38. doi:10.1093/nar/gkab951 PMC8728233 34718731

