# Supplementary material for: Diversity of Antarctic sea ice and under-ice seawater RNA viruses
Source: Appl Environ Microbiol. 2026 Aug 26;92(9):e01472-26. doi: 10.1128/aem.01472-26 (PMC13599536; doi:10.1128/aem.01472-26)
Supplement: Supplementary file 1 [file aem.01472-26-s0001.pdf]

Supplementary Materials for

Diversity of Antarctic Sea Ice and Under-ice Seawater RNA viruses

Shujuan Sun *et al.*

*Yantao Liang. Email: liangyantao@ouc.edu.cn;

*Chen Gao. Email: gaochen@ouc.edu.cn;

*Andrew McMinn. Email: andrew.mcminn@utas.edu.au;

*Min Wang. Email: mingwang@ouc.edu.cn;

This PDF file includes:

Fig. S1
Fig. S2

Supplementary Figure

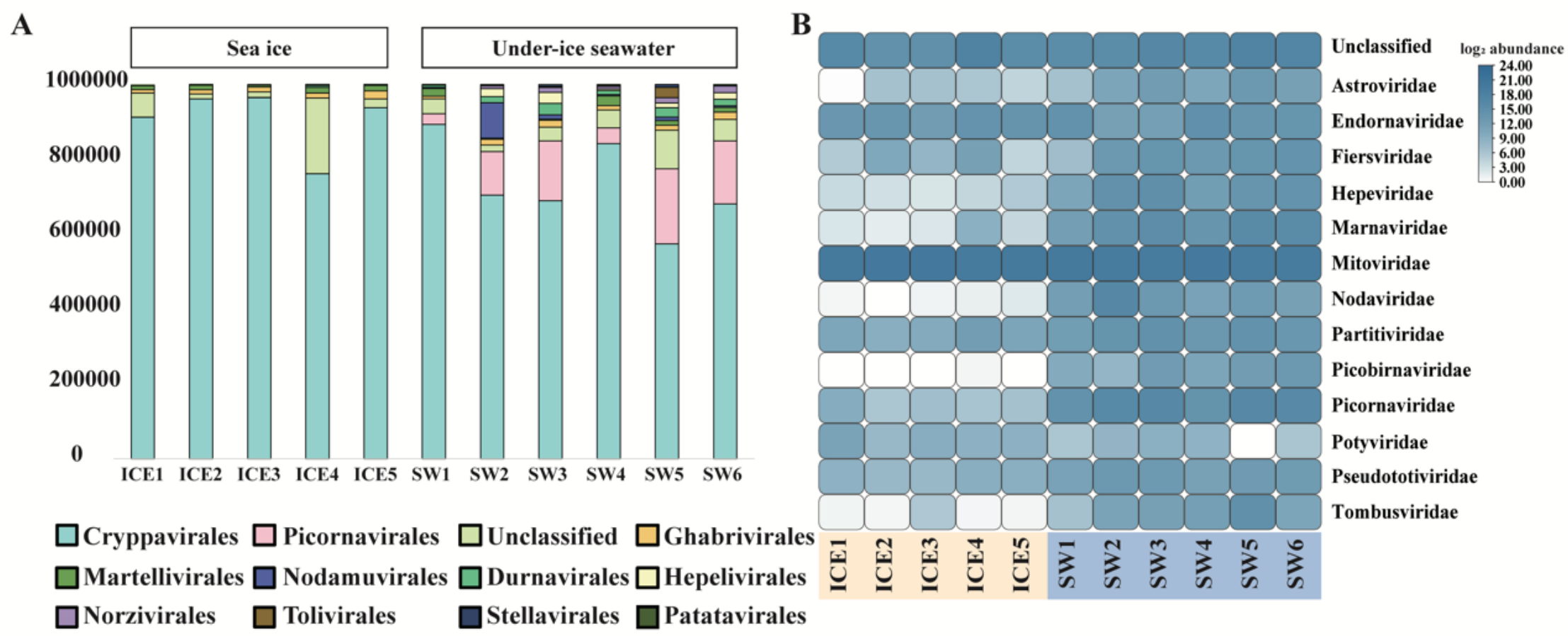

Fig. S1 | Overview of ICE and SW samples virus metatranscriptomic datasets. (A). Based on the relative abundance of viruses, the bar graphs show the number and classification (based on order level) of viral populations in each sample. **(B).** The coverage of abundance for all viral families annotated.

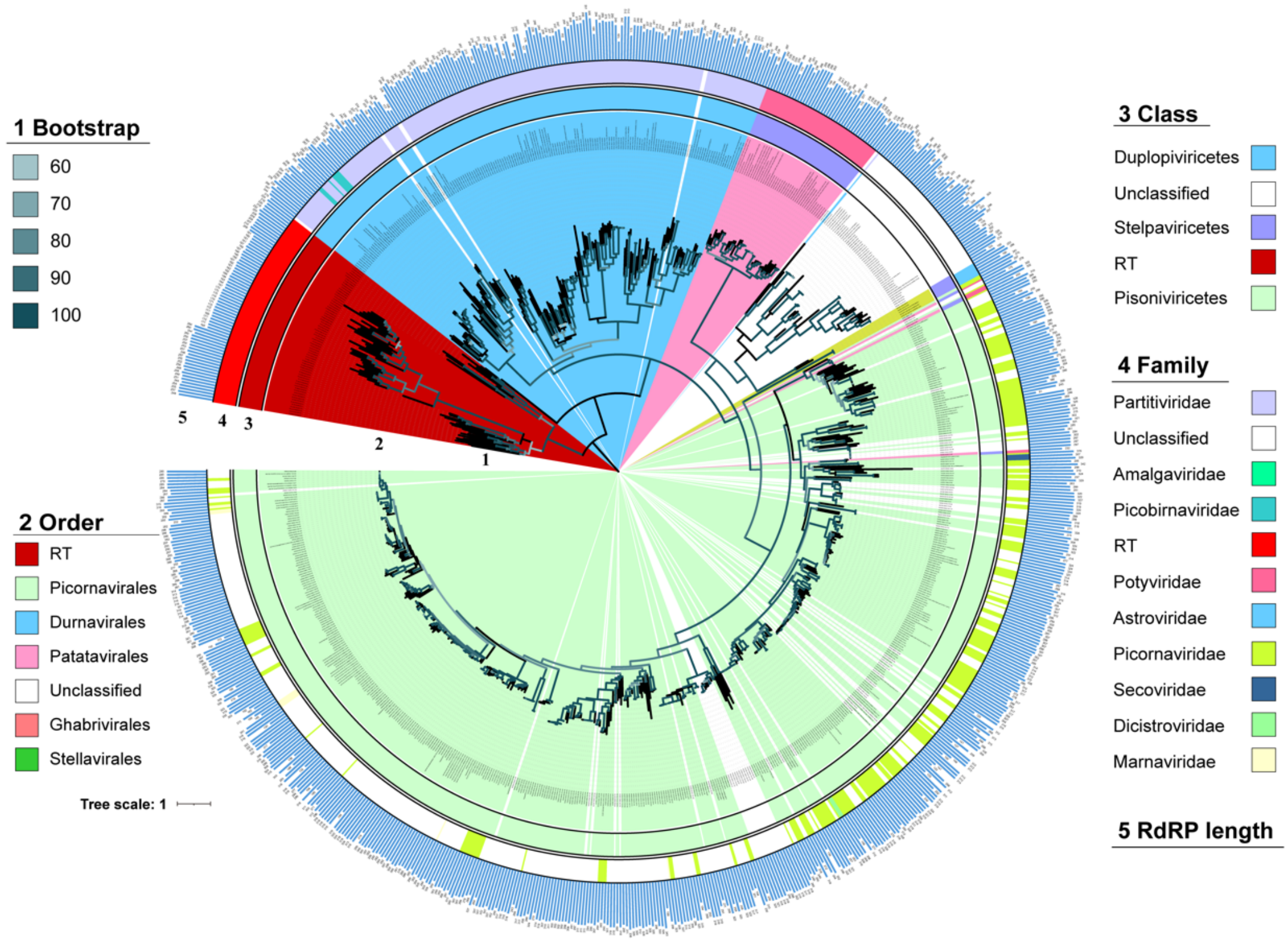

Fig. S2 | Phylogenetic analysis of RdRP. The *Pisuviricota* RdRP maximum likelihood tree was visualized using reverse transcriptase as an outgroup and fixed root with the help of iTOL. The colors of phylogenetic tree branches represent bootstrap values. The inner ring (2) indicates the annotated order, the middle ring (3) and (4) indicates annotated class and family, the outermost ring (5) indicates the length of each RdRP sequence.
